# A new comprehensive oral health literacy scale: development and psychometric evaluation

**DOI:** 10.1186/s12903-021-01795-7

**Published:** 2021-09-05

**Authors:** Yue Sun, Jing Sun, Yan Zhao, Aixiao Cheng, Junhong Zhou

**Affiliations:** 1 Department of Nursing, Peking University First Hospital, Beijing, China; 2 Department of Community Nursing, School of Nursing, Peking University, 38 Xueyuan Rd, Haidian District, Beijing, China; 3 The School of Biomedicine, Beijing City University, Beijing, China; 4 Department of Stomatology, Beijing Zhongguancun Hospital, Beijing, China

**Keywords:** Oral health literacy, Instrument, Reliability, Validity

## Abstract

**Background:**

It has been widely accepted that oral health status is related to oral health literacy. The need to measure oral health literacy has led to the development of measurement instruments. This study aimed to develop a comprehensive instrument for adults and to examine its reliability and validity in China.

**Methods:**

A three-step design process was used. First, a literature review and expert panel discussion were used to draw up a 37-item pool covering oral health knowledge, belief, practice, skill, and functional oral health literacy. The Delphi method was used to delete and modify questions in the item pool. The draft instrument was evaluated by nine experts and the consensus among them was calculated using the content validity index. The scale was then used to conduct a psychometric study among 370 participants from community health centers in Beijing. Construct validity, discriminant validity and concurrent validity were examined. The Cronbach’s alpha coefficient, and test–retest methods were used to assess reliability.

**Results:**

The final scale included 30 items across four dimensions. The item–level content validity index was 0.90. Exploratory factor analysis extracted four fixed factors, and the result of the Kaiser–Meyer–Olkin and Bartlett’s tests was 0.752, with the model explaining 35.21% of the total variance. The four dimensions were associated with oral health knowledge, perceptions of oral health issues, oral health practice and skills, and functional oral health literacy. The mean score of the lowest 27% was significantly lower than the highest 27% (*P* < 0.01), suggesting adequate discriminant validity. The associations between comprehensive oral health literacy scores and educational level, income and self-reported literacy level were significant (*P* < 0.001), showing adequate overall concurrent validity. Internal consistency and test–retest reliability were acceptable, with a Cronbach’s alpha of 0.72 and a total test–retest reliability coefficient of 0.979.

**Conclusions:**

Initial testing of the comprehensive oral health literacy instrument suggested that it is a valid and reliable instrument to evaluate individuals’ oral health literacy, with four dimensions for evaluating knowledge, belief, skills, and functional oral health literacy.

## Background

In 1998, the World Health Organization (WHO) defined health literacy (HL) as “cognitive and social skills that determine an individual’s motivation and ability to acquire, understand, apply information and promote and maintain health through these skills” [1]. Helitzer argued that other HL dimensions should be assessed, because knowledge and attitudes about a health issue affect ability and desire to participate in personal care [2]. HL is therefore multidimensional and the concept has evolved considerably over time [3]. Good HL needs (1) reading, writing, and calculation skills; (2) basic knowledge about health promotion and disease prevention; (3) health awareness and scientific belief about health issues; (4) ability to implement healthy practice in a way that promotes health; and (5) ability to apply scientific methods, deal with health problems, and avoid disease risk factors [2, 3].

Oral health literacy (OHL) is an important branch of health literacy and has an important influence on oral health [4]. It was first defined in the US Department of Health and Human Services policy, *Healthy People 2010*, as the “degree to which individuals have the capacity to obtain, process and understand basic oral health information and services needed to make appropriate health decisions” [5]. Naghibi argued that OHL is a general term that encompasses reading, writing, numeracy, speaking, listening, and proper decision-making skills [6]. The Ismail OHL framework indicated that oral health knowledge, oral health practice and self-efficacy are important components of OHL [7]. OHL is therefore a multidimensional and evolving concept.

Oral health is a key indicator of overall health, well-being and quality of life, as WHO stated [8]. Many studies have shown that oral health status is related to OHL [9, 10]. Baskaradoss showed that more than a third of people with limited OHL had high periodontal risk levels, compared with about 7% of those with adequate OHL [10]. Patients with low OHL have difficulty understanding the importance of health instructions or preventive dental procedures. Good OHL is therefore essential to improve awareness of oral disease and knowledge about methods of disease prevention and health maintenance, and to increase desirable attitude and practice [11].

A scoping review of existing OHL tools found that instruments to evaluate OHL mainly use two strategies: word recognition (REALD-99, REALD-30, REALM-D and REALMD-20) and reading comprehension (TOFHLiD and HKOHLAT-P) [12]. An alternative, the oral health knowledge test (CMOHK), aims to assess OHL levels through measurement of conceptual oral health knowledge [13]. Later developments included relatively comprehensive oral health tools, such as OHLI [14] and OHL-AQ [6], which contain a valid measure of functional oral health literacy (FOHL) encompassing reading, writing, numeracy, speaking, listening and decision-making skills. These more comprehensive measurement tools enriched knowledge of OHL, but the stability of these scales and their applicable populations requires further research [6].

OHL is a multi-dimensional concept, but there is no evaluation tool that covers all its known elements [7]. This study therefore attempted to develop and psychometrically evaluate a comprehensive instrument to measure OHL among adults in China. It aimed to encompass five dimensions of OHL: (1) FOHL (reading, writing, calculation skills and ability to make appropriate health decisions); (2) oral health knowledge (basic knowledge about oral health); (3) oral health beliefs (health awareness and scientific attitude towards health issues); (4) oral health practice (ability to implement healthy practice appropriately); and (5) oral health skills (applying scientific methods and having the necessary ability to deal with health problems).

## Method

### Conceptual framework

The concept of HL and the Ismail OHL framework [7] were used as the framework for the development of a comprehensive oral health literacy (COHL) instrument. Both HL and OHL are multidimensional concepts. The new instrument was therefore conceptualized as needing to capture oral health knowledge, belief, practice, skill and FOHL, the five hypothesized dimensions of COHL.

### Item generation

**Fig. 1:**
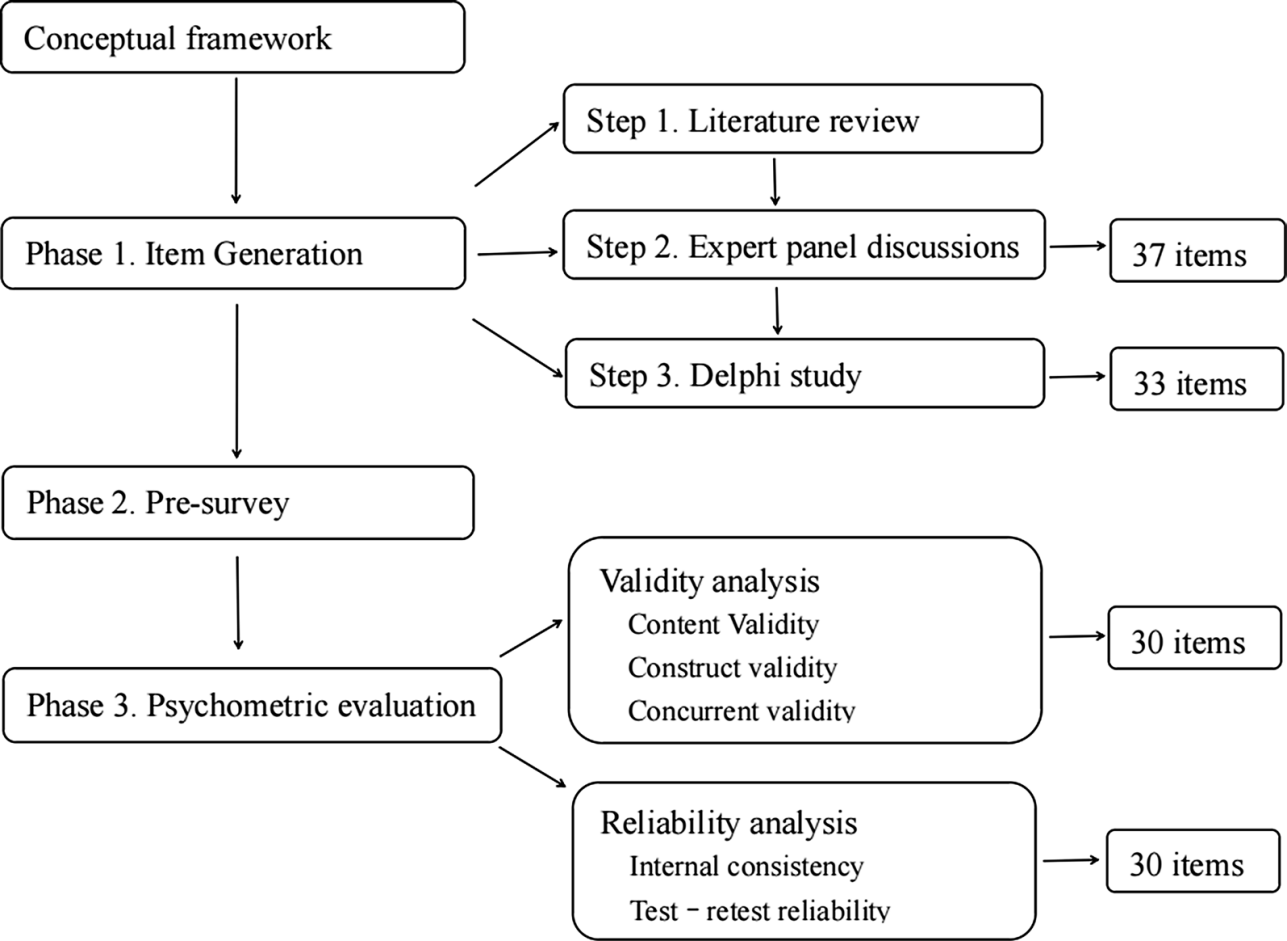
Flowchart of the development and psychometric evaluation design phases of the COHL

The comprehensive oral health literacy (COHL) instrument was created in several phases (see Fig. 1).

### Literature review

A thorough literature search was used to develop an item pool, drawing on PubMed, Web of Science, The Cochrane Library, CNKI and Wanfang to identify concepts, components, and scales for OHL. We consulted guidelines, health education leaflets and information sheets for patients to ensure that all elements were included.

The OHL-AQ scale [6] formed the basis of the COHL scale. This is a valid and reliable instrument for the functional assessment of adults’ OHL [15]. We obtained consent from the scale’s author to translate it into Chinese. We followed the WHO’s translation principles [16]. A bilingual panel of experts, including the original translator, public health experts and experts with experience in translation and creation of assessment tools, reached a consensus about the translated version of OHL-AQ and eliminated any differences. The initial Chinese version was back-translated into English by an independent Chinese translator who was not familiar with the questionnaire. The back-translated English version was cross-matched with the original OHL-AQ.

### Expert panel discussions

Expert panel discussions were held to establish the initial instrument, involving seven experts in oral diseases, oral nursing, epidemiology, and community medicine. After the panel discussion, the initial instrument was formed, including two sections: the OHL assessment and demographic information. The OHL assessment section included 37 items covering knowledge (six items, Knowledge 1–Knowledge 6), belief (six items, Belief 1–Belief 6), practice (seven items, Practice 1–Practice 7), skills (five items, Skill 1–Skill 5), and FOHL (13 items, FOHL 1–FOHL 13). Demographic information section included gender, age, marital status, educational level, monthly income, household composition, smoking status, any chronic conditions and oral health self-assessment. Then the OHL assessment section was conducted in the Delphi study and psychometric evaluation.

### Delphi study

The Delphi method was used to modify the items further. A further nine experts (three oral health specialists, three oral nursing experts, one public health expert, one health educator, and one methodologist) with varying years of experience were invited to the consultation. They were given relevant information, including a brief introduction to the research and the current dimensions and items. In each round, they were asked to determine the relevance of each item using a five-point Likert-type scale, with a score range of 5 (most relevant to COHL) to 1 (least relevant) [17]. They were also asked to evaluate the clarity and simplicity of each item by choosing Yes or No. They could propose other amendments, and if they did so, were asked to provide comments or justifications. We then developed a consultation questionnaire, which was sent to the experts by e-mail, and they were asked to provide feedback within a prescribed time. The dimensions, items and some of the language used were amended following their suggestions.

### Scoring criteria

In the items related to the “knowledge”, “practice” and “skill” domain, correct responses were given a score of one, and incorrect and don’t know responses or blanks were given a score of zero. The “belief” section had a score of 1 for agree and 0 for disagree or no idea [18]. For multiple-choice questions, 1 point was given for correctly choosing ≥ 60% of the correct options, otherwise, 0 was given [19]. Finally, items related to the FOHL domain were scored using the OHL-AQ scale score criteria.

### Pre-survey

A small sample of the target audience (20 participants) completed the questionnaire before recruiting the final sample. This provided feedback on topics such as clarity and understanding of items [20]. This step: (1) verified that the instructions given were easy to follow; (2) established how long it would take to complete the questionnaire; and (3) allowed face validity to be assessed [21]. A reliable technique for assessing face validity is the “think out” model [22], in which participants verbally described their thought processes as they completed each item. A focus group interview between researchers and participants was then conducted with questions such as “What do you think this section is testing?”, “Are you unfamiliar with any of the terms used in this question?”, and “Do you find this question confusing or intentionally misleading?” After two rounds of these steps, we made some changes to terminology and wording of items to reflect feedback. For example, we replaced ‘saprodontia’ with ‘decayed tooth’; ‘gingival’ with ‘gum’ and ‘visit your dentist every six months’ with ‘visit your dentist regularly’.

### Psychometric evaluation

#### Participant recruitment

A convenience sampling method was used to recruit participants from 17 locations in Beijing, China (Zhongguancun, Xinjiekou and Jimenli community health service centers and 14 affiliated community health service stations), between January and April 2020. Outpatients and community residents who attended the monthly health lecture were selected. Potential participants met with trained research assistants after signing to give informed consent. The size of the sample was determined by the number of items in the scale. The sample size should be five to ten times the number of items [23]. There were 33 items in the adjusted scale, ten times of the items was 330, and increased by 10% to allow for invalid responses, 370 participants were eventually recruited. The sample inclusion criteria were: (a) 18 years old or over, (b) living in Beijing; (c) having normal communication ability; and (d) giving written informed consent. Exclusion criteria were having cognitive impairment or mental health problems.

#### Data collection

The questionnaire was filled in by the respondents themselves or by researchers during a face-to-face interview when necessary. The questionnaires were collected on site, and evaluated for missing data. If necessary, the questionnaire was returned to be completed by the respondents.

### Statistical analysis

The database was built using Epidata3.0 software, and data were analyzed using SPSS 21.0. Continuous variables were shown as mean and standard deviation (SD), and categorical variables as numbers and frequency. The psychometric properties of the new scale were measured using validity and reliability.

#### Content validity

Content validity is the degree to which the items of an instrument fully reflect the construct [24].The content validity in this study was assessed using the content validity index (CVI) in the final round of Delphi [25]. The CVI for an item is the proportion of experts who rate it as 4 or 5 [26]. Items for which the CVI exceeded 0.8 were considered sufficiently relevant to OHL [27], and consensus among the judges indicated high content validity.

#### Construct validity

Exploratory factor analysis was used to examine the construct validity of the scale. If the Kaiser–Meyer–Olkin (KMO) value was < 0.6 [28], and Bartlett’s test of sphericity was not significant (*P* > 0.05) [29], the sample data were considered unsuitable for factor analysis. Principal component factor analysis was used to determine the common factors. Five common factors were extracted using the maximum variance rotation method, and the output was the rotation solution. If the item–factor loadings were < 0.40, this item was deleted [30].

#### Discriminant validity

Discriminant validity is the ability to distinguish two or more distinct groups [31].Every participant was given a total score for the questionnaire, and two independent sample *t*-tests were used to compare the highest- and lowest-scoring 27% of the samples (PH and PL) to test the discriminative efficiency of the scale [31]. If *P* < 0.05, the scale was considered to have discriminant validity.

#### Concurrent validity

Concurrent validity assesses the ability of the instrument to distinguish between groups that it should, theoretically, be able to distinguish [32]. Concurrent validity was measured by comparing COHL scores across categories of ages, education level, frequency of visits and oral condition self-assessment [14]. We hypothesized that people with poor COHL were likely to be older, less educated, with lower income and poorer self-assessment of their oral health. Differences in means were tested using the Kruskal–Wallis test, because the scores for COHL were not normally distributed.

#### Internal consistency

The internal consistency was judged using Cronbach’s alpha coefficient and split-half reliability. It was considered unacceptable if the Cronbach’s alpha coefficient was lower than 0.60 [21].

#### Test–retest reliability

The test–retest analysis asked 30 individuals to complete the scale again after 15 days [28]. These participants came to the community health service center again after an interval of 2 weeks. The test–retest reliability was considered acceptable when the correlation coefficient was higher than 0.75 [27].

All above methods were visually illustrated in Fig. 1.

## Results

A total of 370 people participated in testing the scale (see Table 1 for the general characteristics of the sample), of whom 64% were women. The mean age was 46 ± 18 years, with a range of 18–86 years. More than half of the sample had university degrees or above. Most of the remainder attended junior college. The mean monthly income was more than ¥6000. Only a fifth of participants rated their oral health as good, and over 60% rated it as average. Nearly 71% had a chronic disease, often hypertension, and 55.9% said that they had obtained their oral health knowledge from medical workers.

**Table 1:** Sociodemographic characteristics of the study participants (N = 370)

	n (%) or mean ± SD	%
Age (range 18–86 years)	46 ± 18	/
Gender		
Male	133	35.9
Female	237	64.1
Educational level		
Less than primary school	6	1.6
Junior high school	16	4.3
Senior high school or technical secondary school	37	10.0
Junior college	79	21.4
College/university diploma or higher	232	62.7
Monthly income (RMB yuan)		
< 2000	18	4.9
< 4000	37	10.0
< 6000	84	22.7
< 8000	80	21.6
< 10,000	43	11.6
≥ 10,000	108	29.2
Living with		
Live alone	69	18.5
Spouse	228	61.7
Children	40	10.9
Other caregivers	33	8.9
Smoking		
Yes	35	9.5
No	335	90.5
Chronic diseases		
No	108	29.2
Hypertension	66	17.8
Hyperlipidemia	45	12.2
Diabetes	29	7.8
Cardiovascular and cerebrovascular diseases	33	8.9
Oral condition self-assessment		
Good	78	21.1
Moderate	233	63.0
Poor	59	15.9

### Content validity

In the first Delphi round, there was no change in dimensions. However, there were 4 items with I-CVI values of < 0.8, so Knowledge 6 (What is the most important preventive measure for root caries?), Practice 1 (How many times do you brush your teeth every day?), Practice 2 (How do you place your toothbrush?) and Skill 5 (How should you protect dentures in daily life?) were deleted, leaving 33 items. Four items were therefore removed, leaving 33 items. In the second round, the CVI was above 0.80 for each item, and the average CVI for all items was 0.90. There was therefore no change in dimensions and items. The wording of two items was improved following feedback. After two rounds, the opinions of the experts tended to be consistent, indicating that the filtering of dimensions and items was complete. Therefore, the scale included demographic data and 33 items covering knowledge (five items), belief (six items), practice (five items), skill (four items) and FOHL (13 items).

### Construct validity

The KMO coefficient was 0.752, and the Bartlett's test of sphericity was significant at the advanced level (χ^2^ = 2236.0; *P* < 0.001). The sample size was suitable and sufficient for factor analysis. As shown in Table 2, the principal component factor analysis with varimax rotation yielded a four-factor solution. The values of factor loading greater than 0.4 were bold. FOHL 3 (What should you do if some bleeding occurs after brushing or flossing?), FOHL 4 (What should you do if you have pain in your mouth?) and FOHL 10 (Should you swallow mouthwash?) were removed. The four sub-dimensions were considered to explain 35.21% of the total variance.

**Table 2:** Factor loading from the exploratory factor analysis of the COHL (N = 370)

Items		Factor1	Factor2	Factor3	Factor4
*Functional oral health literacy*					
F8	If you take the first capsule at 2 P.M., when should you take the next one? By prescription, take one capsule three times (every 8 h) a day	**0.657**	0.031	− 0.067	− 0.049
F9	If your symptoms are gone by the 4th day of taking the medication, should you stop taking the medication?	**0.626**	− 0.010	0.134	− 0.069
F11	If you use sodium mouth rinse at 12 A.M., when can you eat or drink?	**0.608**	− 0.008	− 0.076	0.018
F12	If your tooth was extracted at 8 A.M., when should you take the gauze out of your mouth?	**0.537**	0.003	− 0.008	− 0.036
F2	Which is the correct name for the 32 teeth that each person has?	**0.522**	0.229	0.150	0.068
F13	If your tooth was extracted at 8 A.M., can you eat hot food at 2 P.M.?	**0.519**	0.041	0.036	− 0.061
F6	What is the meaning of “I exonerate my dentist from unintentional complications of treatment” in your opinion?	**0.455**	0.223	0.008	0.086
F1	What kind of toothpaste can you use to prevent tooth decay?	**0.424**	0.194	0.212	0.236
F5	Which of the following is the best way to remove stain and calculus from teeth?	**0.419**	0.240	0.130	0.294
F7	What is the meaning of “I have a history of allergy to some drugs” in your opinion?	**0.413**	− 0.040	− 0.065	0.163
*Oral health knowledge*					
K3	Which of the following are detrimental factors to oral health?	0.087	**0.738**	− 0.094	0.061
K5	Which of the following are the symptoms and manifestations of periodontal disease?	0.079	**0.684**	0.079	− 0.050
K1	Which of the following are signs of poor oral health?	0.267	**0.675**	− 0.140	0.084
K2	Which of the following behaviors are good for oral health?	0.258	**0.652**	− 0.023	0.089
K4	Which of the following health problems are associated with oral health?	− 0.040	**0.448**	0.169	− 0.031
*Oral health skill*					
S2	How long and what strength when you brush your teeth?	0.170	− 0.030	**0.600**	− 0.033
S3	How do you use dental floss?	0.215	0.113	**0.584**	0.040
S4	Do you use interdental brush every day?	0.017	− 0.164	**0.528**	− 0.037
P4	Do you floss every day?	− 0.103	0.205	**0.485**	0.015
P6	How often do you usually go to the hospital to have your teeth cleaned?	0.037	− 0.067	**0.472**	0.056
S1	What kind of toothbrush would you choose to use?	0.308	0.061	**0.472**	0.189
P3	Do you gargle after each meal?	− 0.184	0.118	**0.447**	0.032
P5	How often do you change your toothbrush?	− 0.095	0.158	**0.433**	0.127
P7	If you have any of the following situations, would you go to the hospital for treatment?	− 0.083	0.598	**0.222**	0.071
*Oral health belief*					
B4	Do you think oral diseases can be prevented?	0.245	0.176	0.076	**0.663**
B5	Do you think you should go to the hospital if you have oral problems?	0.192	0.075	0.046	**0.625**
B1	Do you think oral health is important?	− 0.137	− 0.024	− 0.057	**0.589**
B6	Do you think it is necessary to improve oral health knowledge by attending lectures and reading books?	− 0.002	0.014	0.101	**0.508**
B2	Do you think it is important to keep your mouth clean and hygienic?	− 0.065	− 0.178	− 0.060	**0.438**
B3	Do you think it is inevitable for you to lose teeth when you are old?	0.042	0.155	0.180	**0.425**

*F* Functional oral health literacy, *K* knowledge, *S* skill, *P* practice, *B* belief

Item number: 30

### Discriminant validity

Table 3 shows the discriminant validity of the scale. Each of the factors and total COHL all had a statistically significant difference between the mean scores of the highest (PH) and lowest (PL) 27% of respondents (*P* < 0.05).

**Table 3:** Discriminant validity of the COHL (N = 370)

	Range of scores	Mean ± SD		t	P*
		Scores of PH	Scores of PL		
Knowledge	0–5	3.4 ± 1.3	1.0 ± 1.4	12.8	< 0.001
Belief	0–6	5.7 ± 0.5	4.5 ± 1.3	8.6	< 0.001
Skill	0–9	5.4 ± 1.8	2.0 ± 1.4	15.1	< 0.001
FOHL	0–10	8.2 ± 1.5	3.5 ± 2.1	18.2	< 0.001
Total	0–30	22.6 ± 1.7	11.0 ± 2.8	35.1	< 0.001

*Obtained from two independent samples t-test

### Concurrent validity

Table 4 shows that the overall performance of the scale indicated good concurrent validity. There was a significant association between the OHL scores and age, educational level, income, and self-reported oral health, which confirmed our hypotheses that these factors are related to COHL in adults. Those who had a college/university diploma had higher mean scores in the test of COHL than those with only a primary school education or less. Those aged 18–44 had significantly higher mean scores than those aged 45 or more. Those with a monthly income above 10,000 RMB scored significantly higher than those with less than 2000 RMB. Finally, the mean scores for those with good oral health were also significantly higher than those with poor oral health.

**Table 4:** Concurrent validity of the COHL (N = 370)

	Mean (SD)	P*
Age (years)		
18–44	18.5 ± 5.0	< 0.001
45–64	16.1 ± 5.2	
65–80	14.5 ± 4.7	
Educational level		
Less than primary school	11.2 ± 4.5	< 0.001
Junior high school	10.7 ± 4.1	
Senior high school or technical secondary school	14.2 ± 5.1	
Junior college	16.5 ± 4.8	
College/university diploma or higher	18.4 ± 4.0	
Monthly income (RMB yuan)		
< 2000	14.0 ± 5.9	0.004
< 4000	16.0 ± 5.1	
< 6000	16.6 ± 5.2	
< 8000	16.7 ± 4.5	
< 10,000	17.8 ± 4.5	
≥ 10,000	18. 4 ± 3.9	
Oral condition self-assessment		
Good	18.2 ± 5.0	0.013
Moderate	17.0 ± 4.7	
Poor	15.9 ± 4.5	

*SD* standard deviation

*Obtained from Kruskal–Wallis test

### Internal consistency

The Cronbach’s alpha coefficient for the entire scale was 0.777, and the results for each factor are shown in Table 5. This suggested that the scale was reliable and had good internal consistency.

**Table 5:** Internal consistency and test–retest reliability of the COHL (N = 370)

	No. of items	Cronbach’s α (n = 370)	Test–retest reliability (n = 30)
Oral Health knowledge	5	0.727	0.894* (*P* = 0.002)
Oral Health belief	6	0.512	0.832* (*P* < 0.001)
Oral Health skill	9	0.623	0.929* (*P* < 0.001)
Functional Oral Health literacy	10	0.737	0.941* (*P* = 0.004)
Total: COHL	30	0.777	0.979* (*P* = 0.003)

*Obtained from Pearson relation analysis

### Test–retest reliability

The total test–retest reliability coefficient after 2 weeks was 0.979, and ranged from 0.832 to 0.941 for individual factors. The consistency between the two measurements was statistically significant (*P* < 0.05) (Table 5). The scale therefore showed excellent test–retest reliability.

## Discussion

The aim of this research was to develop a COHL instrument for adults and assess its reliability and validity in a Chinese population. There were three phases in the process: item generation, pre-survey and psychometric evaluation. In contrast to the majority of instruments that only assess a few aspects of oral health literacy, this instrument is conceptually appealing because it integrates oral health knowledge, practice, belief and FOHL. It also had good basic psychometric properties among a group of Chinese-speaking people in Beijing. It is likely to be valuable to measure OHL across four different dimensions. The final version of the instrument contains 10 items for FOHL (Factor 1), five for oral health knowledge (Factor 2), nine for oral health skills (Factor 3) and six for oral health beliefs (Factor 4). The instrument is therefore considered suitable for future use in this and similar populations.

Conventional methods for appropriate and accurate item pool generation include literature review, focus groups and semi-structured interviews [33, 34]. There is no recognized ‘best practice’ for item pool generation, but literature reviews are widely used [14], and produce reliable findings and a high level of scientific evidence [35]. We used the translation–back-translation procedure for linguistic and cross-cultural adaptation of the OHL-AQ scale, which was similar to other studies [15, 36]. Expert panel discussions were used to identify redundant and inappropriate items, and these were discarded and replaced by equivalent items. The involvement of experts in the panel discussions was an efficient strategy because those involved have experience in the field and can contribute effectively to this process [35]. The item pool was confirmed and reduced to 37 items.

The Delphi technique was used in the content validation process. It is a systematic way of determining the consensus among experts and answering questions that are not subject to experimental and epidemiological methods [37]. The content validity was assessed using the item-level CVI to reflect the adequacy of item sampling [38]. In this study, the average CVI values were 0.9, which is acceptable and is consistent with the findings of MC et al. (CVI = 0.82) and CL et al. (CVI > 0.8) [39, 40], so the remaining 33 questions were considered to be relevant, clear and to cover all topics of OHL.

Exploratory factor analysis was used to identify and confirm the construct validity [28]. Principal component factor analysis with varimax rotation and a scree plot yielded a five-factor solution. On the basis of the maximization of variance and the rationality of explaining the hypothetical framework [41], a four-factor structure was considered most suitable for the COHL, though a five-factor solution was also supported by our hypothetical framework. This is consistent with Zeng Jie Ye’s work on developing a resilience instrument for cancer patients. After the research group discussion, we combined the “practice” and “skill” factors, and the factor analysis results showed that this four-factor model fitted well (Table 2). The factor loads of items F3, F4 and F10 were all less than 0.40, suggesting that they should be deleted [31]. From the item content perspective, F3 and F4 were about decision-making, and were very similar to P7, so deleting them did not affect the comprehensiveness of the scale. The correct response rate for F10 was above 99%. Stucky and colleagues [42] also removed questions about sugar and smoking because both items were answered correctly by 99.5% of the sample. It was therefore considered reasonable to omit F10. This therefore left the 30-item OHL assessment instrument. The factor load value indicated that item P7 should be placed in the “knowledge” factor. In the end, it was considered appropriate for inclusion in the “skill” factor in accordance with its item content [31].

The final scale had good discriminant and concurrent validity. It could therefore help health providers to distinguish between clients with higher and lower OHL and groups with different self-assessed oral health, education levels, monthly income and age [31]. Oral health literacy level is related to oral health status just as the relationship between health literacy and health status [43]. Self-assessment oral health condition might be appropriate and valid variable for testing concurrent validity of COHL for it is an appropriate indicator for general dentate status evaluation [44]. In our study, the higher level of oral health literacy assessed by the COHL was linked to a better oral health self-assessment, which was consistent with previous studies [14, 43, 45]. In addition, Shih et al. argued that older and less well-educated residents tended to have poorer health literacy [46]. Jones indicated that scores for the HeLD scale were associated with self-rated general health and economic barriers [47, 48]. As found in those studies, these results confirmed our hypotheses that these factors are related to oral health literacy among adults.

The COHL scale displayed high internal consistency and good test–retest reliability in our study. The Cronbach’s α for the entire questionnaire was 0.78, which is an acceptable value according to Nunnally [49]. The COHL scale was found to have an acceptable internal consistency (Cronbach’s α = 0.70), which was comparable to pre-validated TOFHLiD [45] (Cronbach’s α = 0.63) and OHLI [14] (Cronbach’s α = 0.85). For the four sub-elements, the Cronbach’s α values were moderate to high (ranging from 0.51 to 0.74), similar to De Bourdeaudhuij’s research results [50]. A high test–retest reliability (0.97) indicates that the questions are understandable and the responses reproducible [51]. The cross-sectional nature of the data means that computation of test–retest reproducibility was an added advantage [15].

Our results therefore suggest that the new scale is a comprehensive instrument to measure OHL among adults, with acceptable reliability and validity.

### Limitation

The study results should be considered in the light of some limitations. We used a convenience sample and some bias may have been introduced because participants were recruited predominantly from areas with larger numbers of research institutes and universities. Our sample therefore had relatively high levels of educational attainment. Further psychological assessment is needed to verify the instrument’s universality in a more representative sample. Future studies should also consider sample heterogeneity to improve scale validity. Educational level is not always a good predictor of literacy, but it does play a role. We plan to assess oral health literacy among a population with more diverse educational attainment in a future study. We also did not categorize COHL scores by OHL ability, so further research is needed to establish new cut-offs for COHL scores.

## Conclusion

This study developed a new COHL instrument with acceptable psychometric properties. The new scale is comprehensive, and we believe that it is a valid and reliable instrument for the assessment of adults’ OHL.

## Data Availability

The data used to generate and support the findings of this study are available from the corresponding author upon request.

## Abbreviations

HL: Health literacy
OHL: Oral health literacy
REALD: Rapid estimate of adult literacy in dentistry
REALMD: Rapid estimate of adult literacy in medicine
TOFHLID: Test of functional health literacy in dentistry
HKOHLAT-P: Hong Kong OHL assessment task for paediatric dentistry
CMOHK: Comprehensive measure of oral health knowledge
OHLI: Oral health literacy instrument
OHL-AQ: Oral health literacy-adult questionnaire
FOHL: Functional oral health literacy
COHL: Comprehensive oral health literacy
CVI: Content validity index
KMO: Kaiser–Meyer–Olkin
